# Supplementary figures and images for: Pulsatile contractions and pattern formation in excitable actomyosin cortex
Source: PLoS Comput Biol. 2022 Mar 30;18(3):e1009981. doi: 10.1371/journal.pcbi.1009981 (PMC9000090; doi:10.1371/journal.pcbi.1009981)

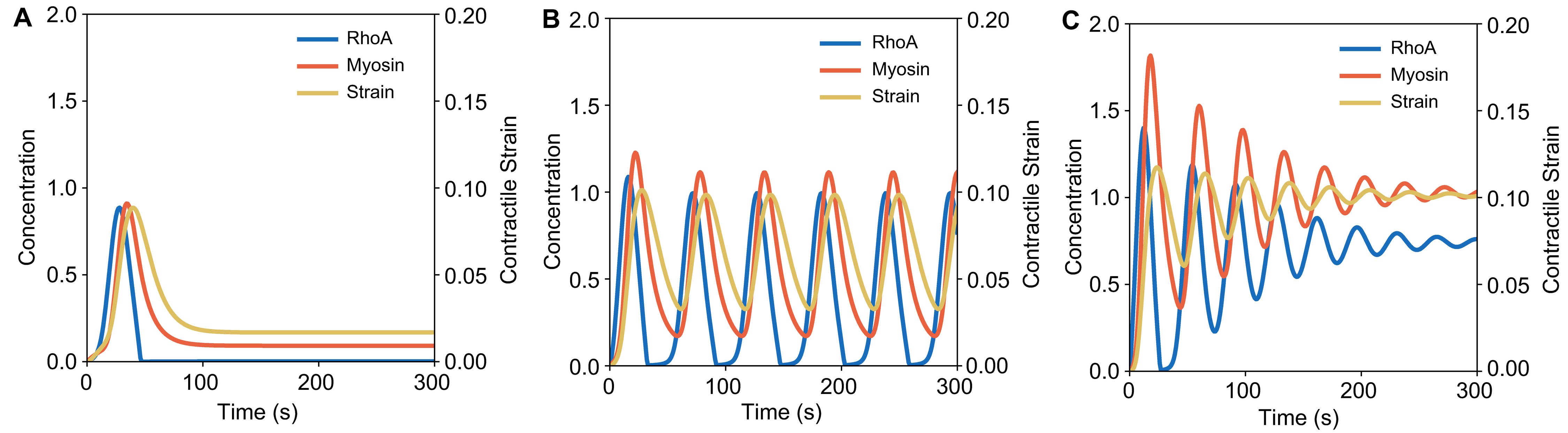

Supplement: S1 Fig — (A-C) Dynamics of RhoA concentration (blue), actomyosin concentration (red), and contractile strain (yellow) in the (A) excitable phase (σa/E = 0.2, S = 0.002 s−1), (B) pulsatile phase (σa/E = 0.2, S = 0.025 s−1), and (C) the contractile phase (σa/E = 0.2, S = 0.075 s−1). (TIF) [file pcbi.1009981.s001.tif]

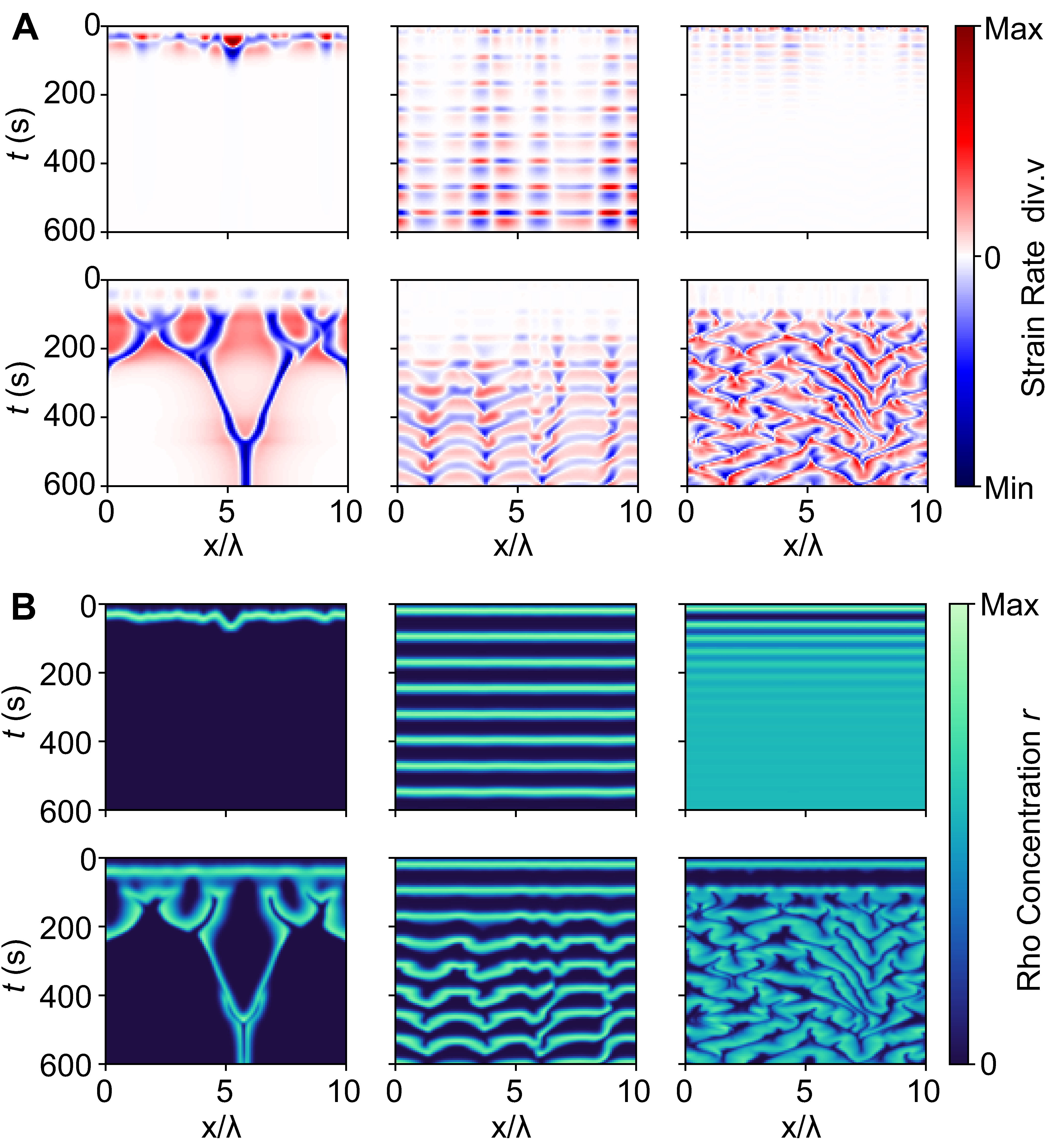

Supplement: S2 Fig — Kymographs of (A) strain rate (∂x v), and (B) RhoA concentration for the different phases in the active gel model corresponding to the actomyosin kymographs shown in Fig 2C. (TIF) [file pcbi.1009981.s002.tif]

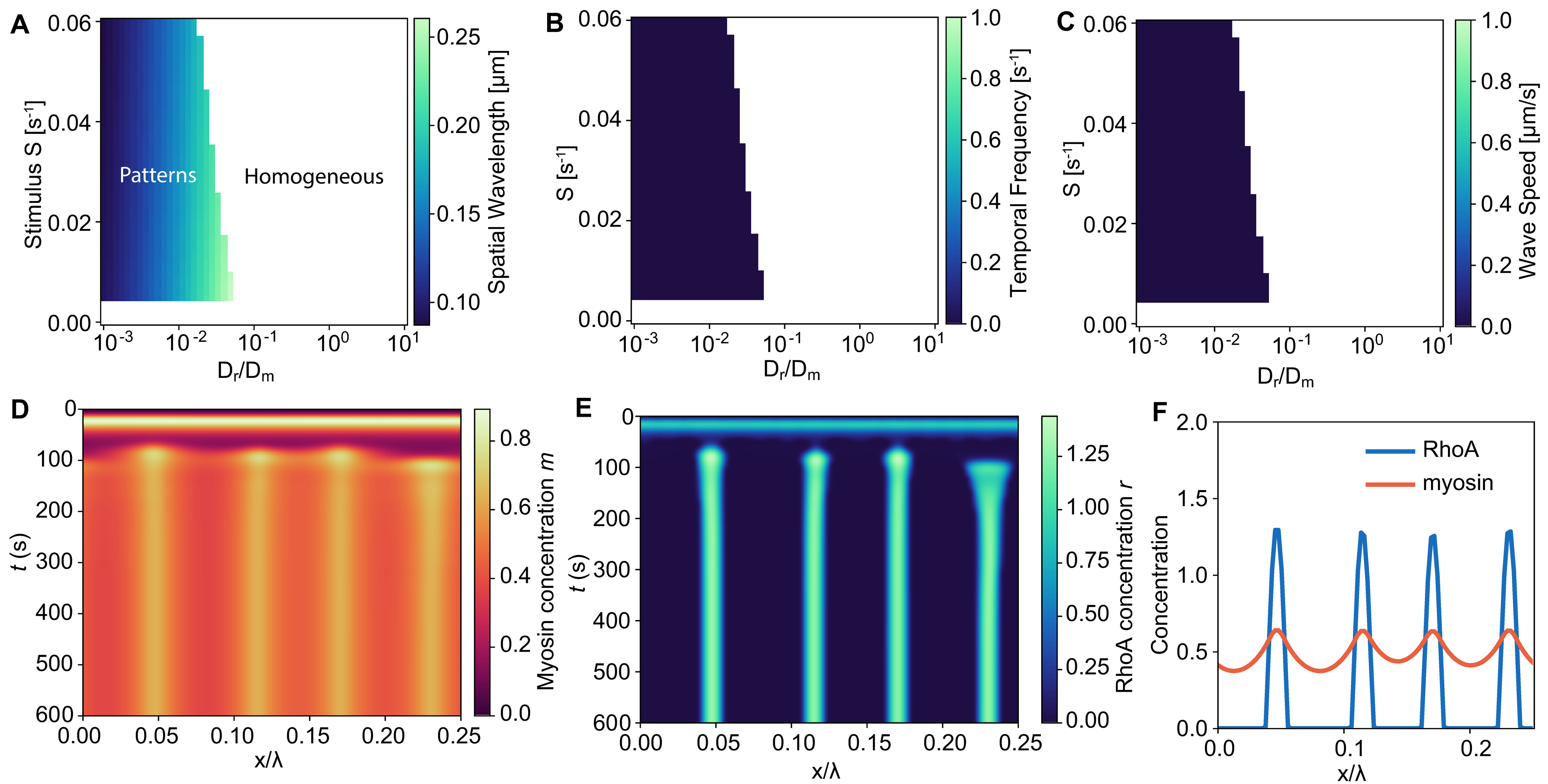

Supplement: S3 Fig — (A) Spatial wavelength, (B) temporal frequency, and (C) wave speed computed by linear stability analysis of the RhoA-myosin reaction-diffusion model with σa′=0, for varying stimulus S and activator to inhibitor diffusivity ratio, Dr/Dm. White regions in the parameter space show homogeneous steady states. Default parameter value of Dr/Dm is 10. (D-E) Kymographs of actomyosin concentration (D) and RhoA concentration (E) showing pattern formation for low RhoA diffusivity (Dr/Dm = 0.01, S = 0.01 s−1). (F) Steady-state spatial profiles of RhoA and actomyosin concentrations for the patterns corresponding to (D) and (E). Overlapping peaks indicates Turing pattern formation. (TIF) [file pcbi.1009981.s003.tif]

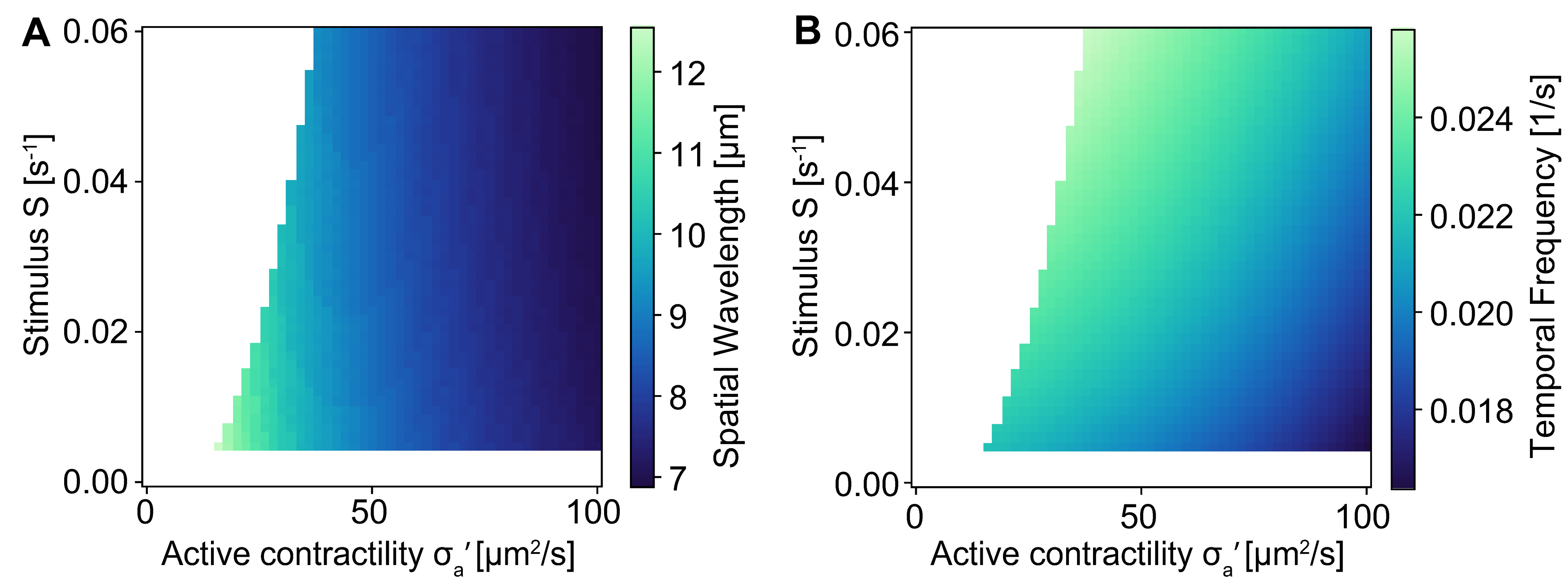

Supplement: S4 Fig — (A-D) Kymographs of actomyosin concentration with (A, C) no RhoA advection, and (B, D) no actomyosin advection. For (A, B) S = 0.00 s−1, σa′=100, and (C, D) S = 0.01 s−1, σa′=100. (TIF) [file pcbi.1009981.s004.tif]

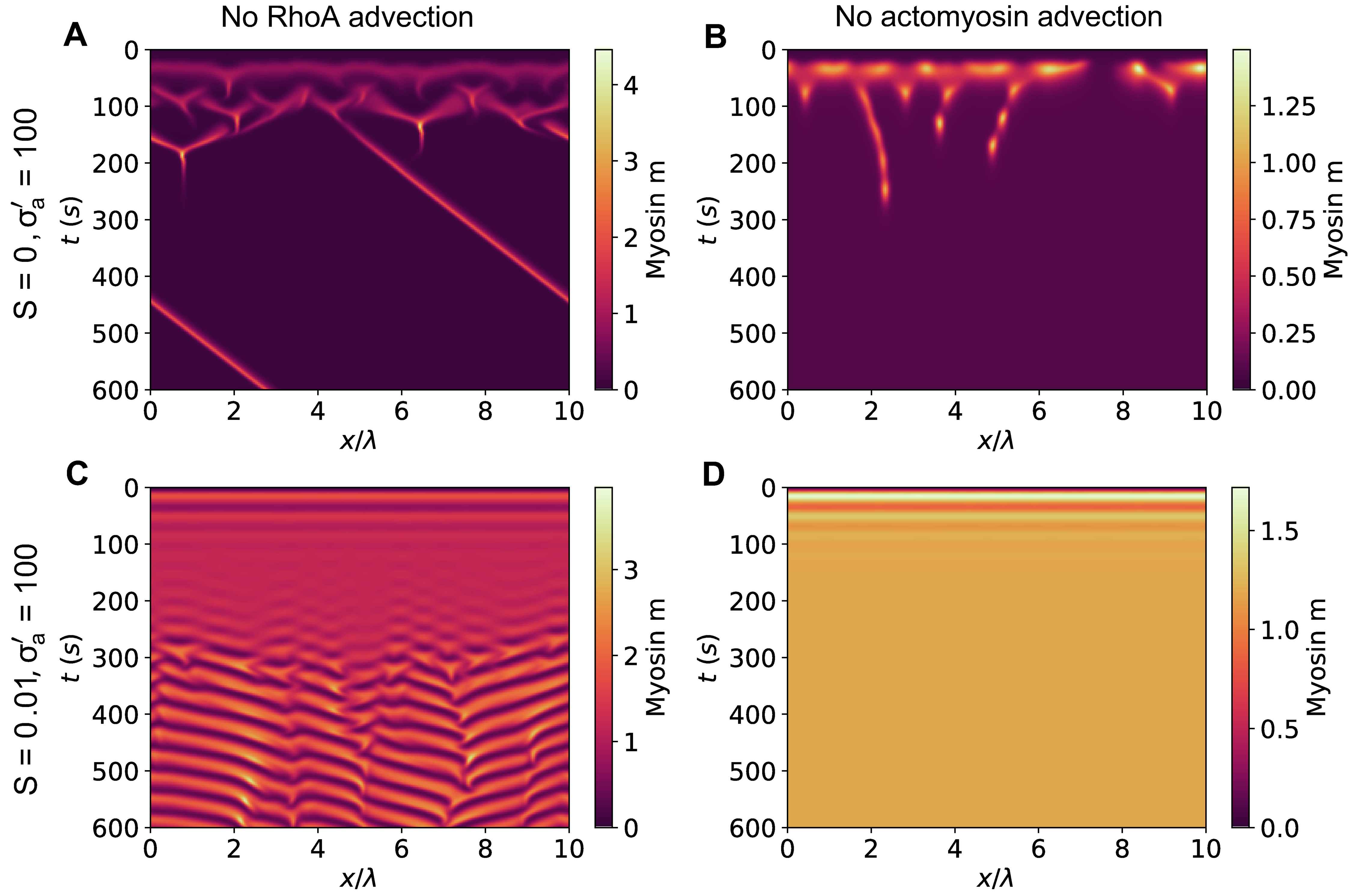

Supplement: S5 Fig — (A) Spatial wavelength, and (B) temporal frequency computed by linear stability analysis for varying active contractility σa′ and RhoA stimulus S. (TIF) [file pcbi.1009981.s005.tif]

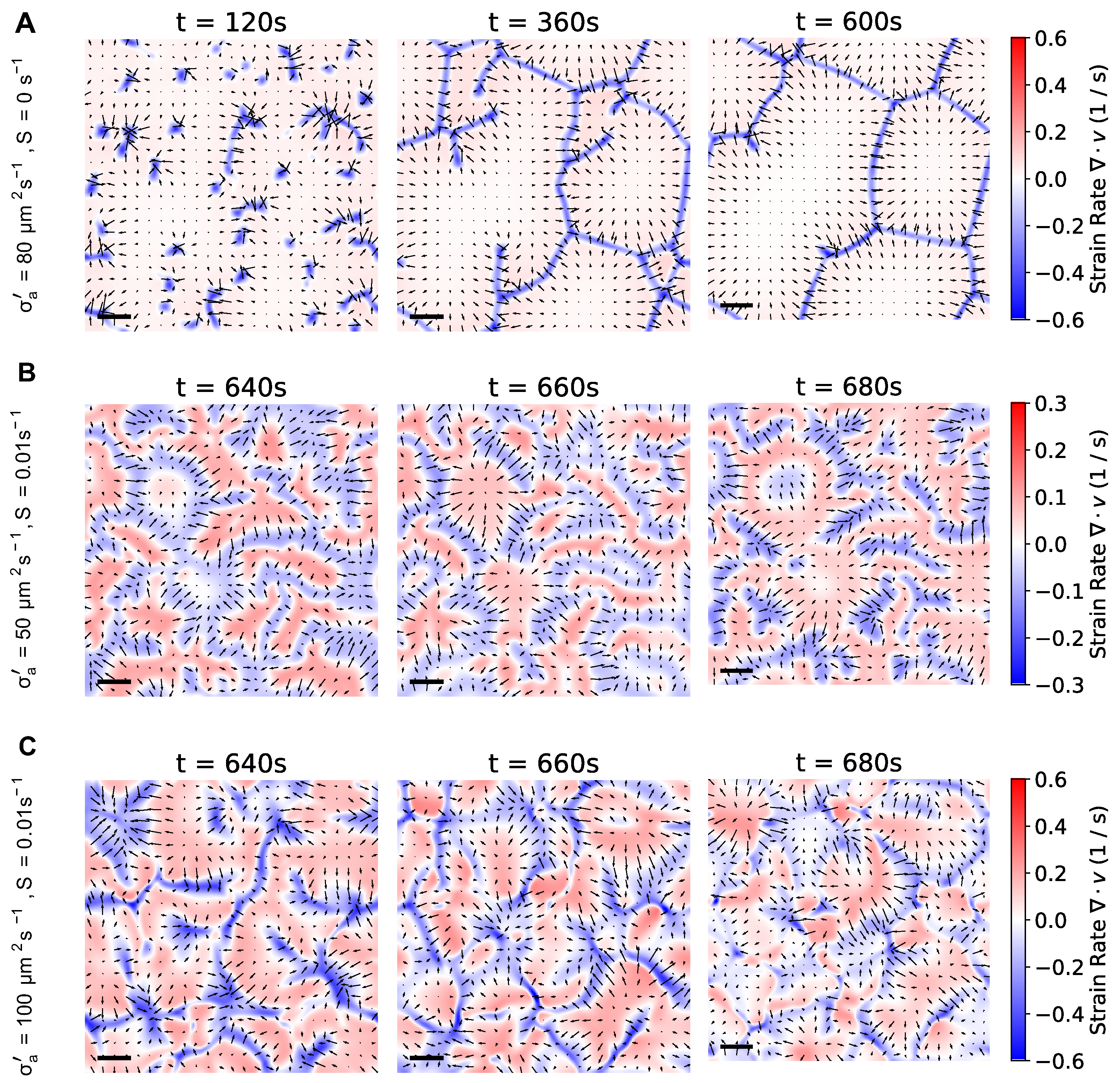

Supplement: S6 Fig — (A-C) Actomyosin concentration field obtained from simulations of the active gel model in two spatial dimensions, showing (A) contractile network formation (σa′=80 μm2/s, S = 0), (B) propagating waves (σa′=50 μm2/s, S = 0.01 s−1) and (C) localized pulsatile contractions (σa′=100 μm2/s, S = 0.01 s−1). Scale bar indicates a distance of λ = 15 μm. Arrows indicate velocity field v and colour the local strain rate ∇⋅v. (TIF) [file pcbi.1009981.s006.tif]
