# Supplementary material for: Pulsatile contractions and pattern formation in excitable actomyosin cortex
Source: PLoS Comput Biol. 2022 Mar 30;18(3):e1009981. doi: 10.1371/journal.pcbi.1009981 (PMC9000090; doi:10.1371/journal.pcbi.1009981)
Supplement: Attachment — Submitted filename: Reviewer Response.pdf [file pcbi.1009981.s011.pdf]

We thank the editors for providing us the opportunity to submit a revised manuscript. We thank all the reviewers for their valuable comments and suggestions that helped us improve our manuscript significantly. We believe that the revised manuscript adequately addresses all the reviewer concerns regarding the clarity of presentation, model assumptions and analyses, as well as comparisons to experiments. Below we provide a point-by-point response to all the reviewer comments and questions.

Reviewer #1

Review on the manuscript "Pulsatile contractions and pattern formation in excitable actomyosin cortex" by M.F. Staddon, E.M. Munro and S. Banerjee submitted for publication to PlosCompBiol.

This is a modelling and simulation study motivated by recent experimental observations [17] on pulsed contractions in the cortex of C. Elegans. The authors formulate a mathematical model for the feedback between RhoA and Actomyosin which has both, a biochemical component (the know Activator-Inhibitor Loop between RhoA and actomyosin) and a mechanical component (the cytoskeleton flow in response to actomyosin contraction).

The study highlights that
1) the activator-inhibitor system alone features a series of different steady state solutions, oscillating patterns and limit cycles depending on parameter values.
2) the spatially inhomogeneous version of this model coupled to contractile flow in one spatial dimension (periodic boundary cond.) features complex spatiotemporal patterns depending on parameters: either oscillating or non-oscillating patterns in time and convective or non-convective patterns in space, as well as any combination of these depending on parameter values.
3) the same observation is made for the model simulated for a 2-dimension spatial domain. The resulting patterns coincide qualitatively well with recent observations for patterns in the cortex of C. Elegans [17] (pulses) and starfish oocyte (propagating waves).

I appreciate that this is a very interesting study using an innovative and beautiful modelling approach which succeeds in explaining qualitatively some of the patterns observed in the cortices of various cell types. This alone I believe already deserves publication as I believe it will pave the way to even more intricate and powerful modelling of actomyosin contraction with realistic cell geometries.

The only concern I have is about the interpretation/parametrisation of the governing equations for the cytoskeleton flows. The derivation relates stress to drag in eq (7). The drag in this expression is taken as independent of the concentration of actomyosin m (the authors lump together the concentrations of F-Actin and of myosin into m). I'd argue that a more appropriate formulation of this relation should involve the density m at least as linear factor in the drag term (more actomyosin -> more drag and vice versa). The way the authors write the equations allows them to derive the governing equation (8) without disturbing additional factors m and m_x, which would probably make it much harder to solve the resulting momentum equation (8) (I guess the velocity field would blow up where m is close to 0). I recognize that probably some sort of regularisation would be needed and omitting m from (7) appears to be the authors' approach of doing that.

We thank the reviewer for their constructive comments, positive appraisal, and for recommending our work for publication in PCB.

Following the reviewer's suggestion, we implemented an actomyosin-dependent friction coefficient, where the friction is a saturating function of actomyosin concentration m: $\gamma = \gamma_0 \left(1 + \frac{m}{m+m_0}\right)$, such

that friction at high actomyosin concentration is double the friction at low concentration. This functional form ensures that friction is non-vanishing at zero concentration. We use a factor of 2 between high and low concentration frictions as an example. We find that the overall phase behaviours of the model remain the same in the presence of actomyosin-dependent friction, as shown by the phase diagram below (similar to Fig 2C). Thus, actomyosin-dependent friction appears to not affect the different dynamic phases observed in our model, but may tune the parameters at which they appear.

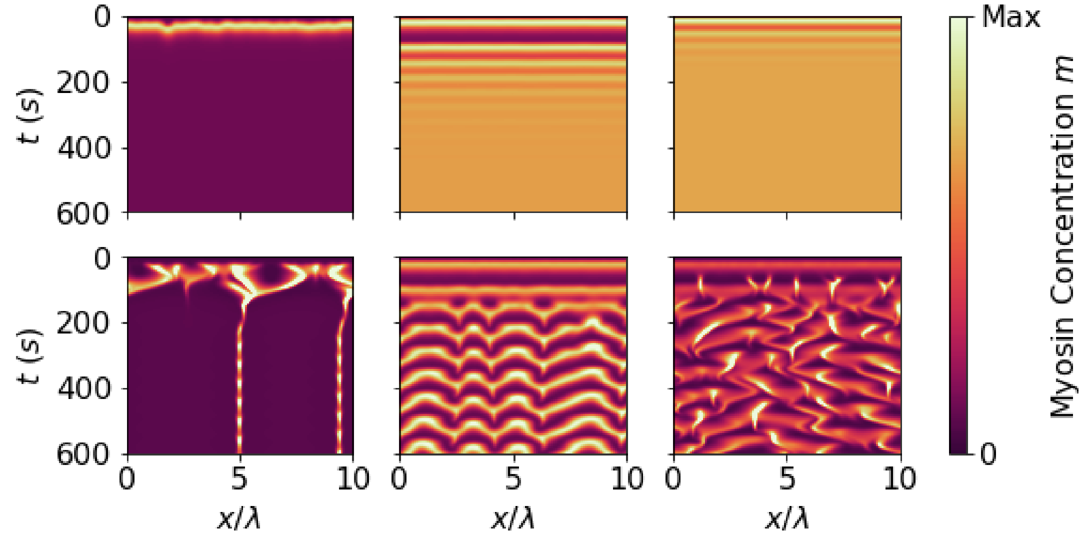

But that approach (assume the cytoskeleton density is constant) is inconsistent with the rest of the modelling, namely that for m a separate continuity equation (10) is stipulated and that according to simulations the value of m ranges between 0 (depending on parameters in most of the domain) and Max. I think all that has to do with not distinguishing between F-Actin concentration and myosin concentration and I believe that this has to be sorted out before publication so the model can be interpreted properly with regards to experimental observations.

We chose not to distinguish between F-actin and myosin concentrations because these are modulated in parallel by different effector pathways (Rho Kinase for myosin and formin/CYK-1 for F-actin) downstream of RhoA. Furthermore, our quantitative multicolor imaging showed that the time course of appearance and disappearance of (normalized) F-actin and myosin intensities measured locally during pulses of RhoA activity are remarkably similar, and the cortical lifetimes of F-actin and myosin II measured by single molecule imaging (Michaux et al, 2018) are also remarkably similar. Thus, it made sense to lump these together as a single species which exerts density-dependent effects on active stress (myosin) and RhoA activity (F-actin). We clarify these points in the revised text.

On that note: it would be good a plot of the simulated velocity field could be added, e.g. to Figure 6 which only show the dynamics of the phase field so far, or to the SM.

We thank the reviewer for this comment. We have now included a supplementary figure (Fig. S6) showing the velocity and strain rate fields corresponding to the 2D simulations showed in Figure 6.

Reviewer #2

The authors present a theoretical study of pattern formation in zero-, one- and two-dimensional systems of excitable actomyosin. The main scheme is presented in Fig. 1a and essentially based on

experimental results from Ref. 17. The central element is RhoA, that is an activator because it promotes its own activity and governs many downstream targets. In contrast to most other work in this field, actomyosin is considered to be one entity only. Most importantly, there is negative feedback from actomyosin back to RhoA, through the GAP RGA-3/4, as described in Ref. 17 for C. elegans. Together, this biochemical system is similar to one of the activator-inhibitor systems envisioned by Turing. In a last step, it is coupled to the mechanics of the system. Here different models are discussed throughout the paper. First we have a very simple model where contractile strain leads to increase in concentration, thereby promoting RhoA and actomyosin. This model has no spatial domain. Later flow and advection are introduced, very similar to work from the Dresden group in Refs. 30 and 32, and considered for 1D and 2D. The authors report a large range of possible behaviour, including quiescence, contraction, pulsations, excitable excursions, solitons, chaos, turbulence. Phase diagrams are provided. The relation to experiments occurs mainly by several comments in the text pointing out similarities to observations in published work.

This work is rather theoretical in nature and in general is an interesting and original contribution to the large field of actomyosin as excitable medium. I especially like the nice progression from simple to more complex models throughout the course of the paper. Yet I also see two major weaknesses. First it is not entirely clear what really is new in regard to theory and how it compares to published work.

We thank the reviewer for their interest in our work and recognizing the originality of our contribution. To address the reviewer's comments, we have now expanded the Discussion section to include more comparisons with previous work, while highlighting the originality of our contribution. Such comparisons are also provided in the Introduction section, as well as throughout the Results section. Previous models of actomyosin cortex dynamics have mainly been either purely biochemical (e.g., Bement et al *Nat Cell Bio* 2015, Kamps et al *Cell Rep* 2020), or based on active gel mechanics (e.g., Mayer et al *Nature* 2010, Nishikawa et al *eLife* 2017). The novelty of our work is combining mechanics and biochemical signalling in a unified theoretical framework, where actomyosin network mechanics and RhoA signalling feedback to each other, regulating the emergent dynamics of the cortex. With this mechanochemical model, we are able to reproduce a number of different observed phases by changing only two control parameters, namely the amount of active stress generated by actomyosin, and the basal rate of RhoA production in the system.

Second the relation to experiments is unclear. Especially for PLOS Computational Biology (in contrast to a pure physics journal) one would like to see a stronger connection to experiments, especially when one of the authors is very well known for such experiments. Below I comment a bit more on both aspects.

The models that we present, and their parametrization, are strongly grounded in experimental data and observations. First, the biochemical circuit that describes interaction between RhoA and actomyosin is inferred from experimental correlations (Michaux et al JCB 2018), and the model parameters are determined from experimentally fitting the model to observed data in the *C. elegans* embryo. These data were presented in Fig. 1b. Second, we couple this biochemical circuit to an active gel model of the actomyosin cortex whose physical properties are parametrized from experimental data presented in Saha et al (2016). We then compare the different phases obtained in our simulations to experimentally observed behaviour in a number of different systems, including *C. elegans* embryo, *Xenopus* and starfish oocytes. While we do not provide any new experimental data, which is not required for publication in PLOS Computational Biology, our modelling builds upon previously published experimental work. According to the scope of PLoS Computational Biology (published in their website), "Inclusion of experimental validation is not required for publication, but should be referenced where possible." We have thus extensively referenced experimental work, wherever appropriate, throughout the manuscript.

Regarding theory work, I feel that much earlier work has to be cited and discussed. Regarding the scheme in Fig. a, the authors should also discuss Kamps, Dominic, et al. "Optogenetic Tuning Reveals Rho Amplification-Dependent Dynamics of a Cell Contraction Signal Network." Cell reports 33.9 (2020): 108467. Their biochemical model is rather similar but more detailed and therefore generates oscillations by itself, without the mechanics.

Thank you for pointing out this relevant paper by Kamps et al, which we have now cited and added to the discussion. We have also been careful to cite other existing literature, wherever appropriate. We would be very grateful if the reviewer could point out any other essential omissions from the reference list.

The purely biochemical model presented by Kamps et al is a bit more detailed than ours by considering the coupled dynamics of GEF, RhoA and Myosin. However, the dynamic behaviour of this biochemical circuit is similar to ours, showing stable, excitable and pulsatile dynamics in different parameter regimes. This model, however, requires stochastic noise in the equations to trigger wave formation due to the excitable nature of the system, which is an additional way for waves to form. Our model can predict spontaneous wave formation and propagation via feedback between mechanics and biochemical signalling which is not included in the model by Kamps et al.

Moreover the negative feedback from myosin to RhoA is another one than mentioned here (inhibition of GEF rather than upregulation of GAP). This leads to the question how specific the model here is to C. elegans and the particular GAP identified here, and which other models should be considered in general? How would the authors' results change when considered the model from Kamps et al. for their biochemistry part?

Studies in echinoderm and frog embryos suggest that in these systems, like *C. elegans*, negative feedback involves delayed inhibition of RhoA activity by F-actin, likely through recruitment of RhoGAP, although this has yet to be established. Thus, the specific form of inhibition we consider here is not specific to *C. elegans* alone. In general, the architecture (i.e., the feedback motifs) of the biochemical circuit that are more important than the specific molecular details in predicting the dynamics of the system. While Kamps et al use a three-component model (Rho, GEF and myosin) with slightly different implementation of negative and positive feedback loops, their system can display both pulsatile and stable regimes as in our model, as does the model in Bement et al (2015). In all these cases, while the molecular details vary, the overall architecture of the biochemical circuit is similar – autocatalytic positive feedback coupled to a negative feedback element. We clarify these points in the revised manuscript.

When diffusion and advection of chemical species are included, with a fast-diffusing activator (RhoA) and slow-diffusing inhibitor (actomyosin), the condition for Turing patterns is not satisfied as the latter requires a fast-diffusing inhibitor and a slow-diffusing activator. We would thus expect spatially uniform states in the absence of noise or additional feedback mechanisms. However, by including feedback with mechanical stresses generated by actomyosin we are able to break the spatial symmetry, resulting in spontaneous formation of patterned states. This stands in contrast to the model presented by Kamps et al, which requires noise to trigger waves and spatial patterns. In a similar sense to Turing patterns that rely only on linear stability of the model to determine the overall response, we should expect only the nature of feedback motifs to matter. We add these discussions to the concluding section of the manuscript.

My second comment is that the effect of concentration increase upon contraction is also not new and has been used for modelling e.g. in this paper: Buttenschön, Andreas, Yue Liu, and Leah Edelstein-

Keshet. "Cell size, mechanical tension, and GTPase signaling in the single cell." Bulletin of mathematical biology 82.2 (2020): 1-33. Concentration effects are also often described in the context of cell growth, when volume is increasing. The authors should discuss better how their model compares with these other models.

Thank you for highlighting this paper by Edelstein-Keshet group. We have now cited Buttenschön et al and have included a brief discussion in relation to our model. Since we do not model a growing cell, we think it is not relevant here to discuss the effects of cell size and growth on the concentration of chemical species. We have considered the effect of cell growth and size on chemical pattern formation in another paper: see Cornwall-Scoones et al *Cells* 9:1646 (2020).

I also miss a discuss of the effect of three dimension: concentration might stay constant because due to the Poisson effect, contraction in one dimension comes with extension in another dimension. Is there experimental evidence that concentration effects are really relevant?

It is generally true that a contraction of a material in one or two dimensions should lead to an expansion in other dimensions. This concern is not relevant for RhoA, which is membrane bound. It is likely that local contraction or dilation can drive variations in the thickness of cortical actomyosin, but how magnitude of cortical stress depends on thickness remains poorly understood and likely depends on details that vary across cells (see e.g., Chugh et al, *Nat Cell Bio* 2017). For this reason, and because there is strong evidence (next paragraph) that stress depends on quasi2D densities of myosin, we could not justify considering 3D effects here. We now discuss this in the main text.

Several recent studies have provided experimental evidence (based on PIV analysis) for the dynamic coupling between actomyosin concentration and contraction, in which local contraction advects and concentrates actomyosin and RhoA (Munjal et al *Nature* 2015, Nishikawa et al *eLife* 2017). There is abundant evidence from previous work in C. elegans (e.g., Munro et al, *Dev Cell* 2004, Mayer et al, *Nature* 2010) that local magnitudes of stresses driving cortical flows depend on quasi-2D densities of cortical myosin (what is observed in experiments). We now cite these papers while discussing concentration effects.

I am a bit confused about the mechanical model used. Eq. 3 looks like Kelvin-Voigt to me, but Eq. 6 is Maxwell. Why do the authors change the viscoelastic model and why is this not explained? Do the concentration effects of the 0D model also exist in the 1D and 2D model?

Thank you for this excellent question. The mechanical model presented in Eq. (3) describes a local contractile element of the cytoskeletal network that is homogeneous in material properties and can support elastic stresses. It is therefore appropriate to model it as a Kelvin-Voigt viscoelastic material, which behaves elastically at long times but dissipates stresses over shorter timescales (via drag with the surrounding medium) with viscosity coefficient η_L. When we expand the model to a 1D (or 2D) continuum, we connect these Kelvin-Voigt elements in series via dashpots that represent longer time scale remodeling of the cytoskeletal network via actomyosin assembly and disassembly with a viscosity coefficient η (see figure below). It is therefore appropriate to model the larger-scale cytoskeletal network as a Maxwell viscoelastic material. These concepts are explained by the schematic below showing the mechanical circuit of the cortical actomyosin network.

In the 1D and the 2D models, the concentration effects come from the advection term. The actomyosin generates contractile stresses that drag in material and locally increase the concentration of RhoA and actomyosin.

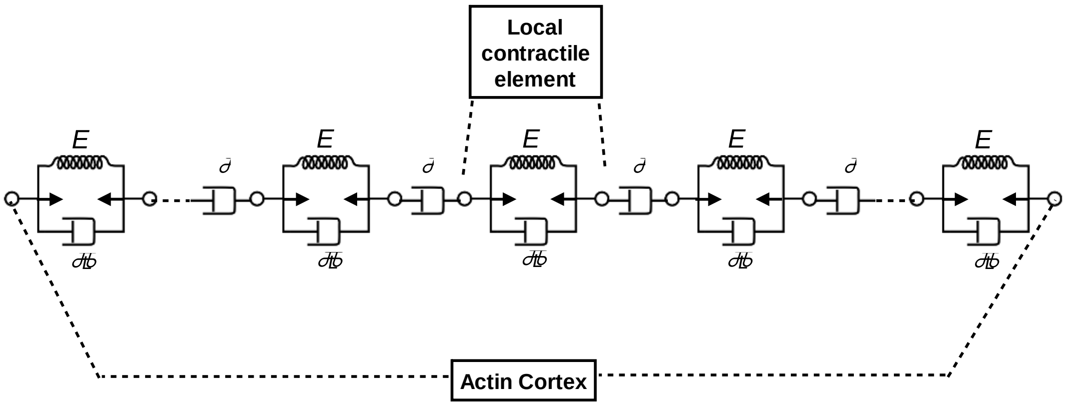

Regarding the phase plane analysis in Fig. 2, I wonder what happens to the third component u. The system is 3D and I do not understand how one can analyze this without the dynamics in u. Is there some kind of adiabatic approximation used here?

The linear stability analysis was indeed performed in 3D, with perturbations applied to RhoA concentration, actomyosin concentration, and the velocity field. The details are included in the supplement.

Recently there has been much work on excitable actomyosin coupled to shape changes, compare e.g. Brinkmann, Felix, et al. "Post-Turing tissue pattern formation: Advent of mechanochemistry." PLoS computational biology 14.7 (2018): e1006259 or the very recent work by the Turlier lab on "A viscous active shell theory of the cell cortex" (https://arxiv.org/abs/2110.12089). Maybe the authors want to comment on their model in this context.

We thank the reviewer for these references, but they do not model excitable actomyosin or the coupling of mechanics with Rho signaling. The paper by Brinkmann et al models a cell (or tissue) as a continuum elastic material. A chemical concentration field induces contraction, and in turn is produced by local strain in a positive feedback loop which can generate patterned contraction and deformations of the cell. We have thus cited the Brinkmann et al paper and added it the discussion as it incorporates effects of mechanochemical coupling. The paper by Turlier group develops 3D continuum model of the cell cortex as a thin elastic shell under active stresses and study the effect of active stress and turnover on cell shape changes. Thus, it is not directly relevant for our work.

I also want to comment that this statement here is not correct: “Travelling waves of actomyosin contraction can propagate across the cortical surface [16, 18].” Such a traveling wave has been described for starfish oocytes in Ref. 20, but not in Ref. 18, where it is explicitly shown that blebbistatin has no effects, so these waves are myosin-independent (in contrast to Ref. 20, where blebbistatin abolishes the wave). The model in that paper works without myosin and the negative feedback in the model is only through actin polymerization. The authors should consider how this fits into their framework and if e.g. the phase analysis for turbulence really depends on the specific model.

We thank the reviewer for this comment and have corrected the citation. We have now added into the discussion the point that waves can be obtained in some cases when the mechanical forces are removed. The model proposed by Bement et al requires stochastic noise to trigger waves, which is an additional mechanism for wave generation in excitable media.

Finally I think that the authors should better explain the relation to experiments. In my view, they should include experimental data going beyond the two curves shown in Fig. 1b. For the bulk of the

paper, there are only theoretical predictions and no direct comparison with experiments, although the authors make a large effort to parametrize with realistic values. For example, how do the correlation lengths from Fig. 5 and 6 correspond to structure formation in the C. elegans cortex? Actually this system shows many aster-like structures and it is not clear if they are predicted here. Can one compare theoretical and experimental flow fields, e.g. after local stimulation? Such comparison would strongly increase impact.

The models that we present, and their parametrization, are strongly grounded in experimental data and observations. First, the biochemical circuit that describes interaction between RhoA and actomyosin is inferred from experimental correlations (Michaux et al JCB 2018), and the model parameters are determined from experimentally fitting the model to observed data in the *C. elegans* embryo. These data were presented in Fig. 1b. Second, we couple this biochemical circuit to an active gel model of the actomyosin cortex whose physical properties are parametrized from experimental data presented in Saha et al (2016). We then compare the different phases obtained in our simulations to experimentally observed behaviour in a number of different systems, including *C. elegans* embryo, *Xenopus* and starfish oocytes. While we do not provide any new experimental data, which is not required for publication in PLOS Computational Biology (see scope), our modelling builds upon previously published experimental work.

Regarding the reviewer's comment on aster-like structures in *C. elegans*, while we do not directly model actomyosin structure in our simulations, the simulated flow fields show aster-like contraction foci (Fig. S6) as seen in experiments (Michaux et al 2018, Nishikawa et al 2017) with length-scales of contraction ~10 μm. Comparing the simulated and the experimental flow fields after local RhoA stimulation would require new experiments with optogenetic tools, which is beyond the scope of this computational/theoretical work. We would like to point out that according to the scope of PLoS Computational Biology, "Inclusion of experimental validation is not required for publication, but should be referenced where possible."

Reviewer #3

1. The feedback loop of activator-inhibitor-strain (Fig. 1) is of course a simplified network extracted from the more elaborate Rho-Rock network. Would the coarse graining implicit in this simplified network not lead to delay terms? If so how would these affect the dynamics.

The intermediate step of ROCK activation will indeed introduce a small time delay. Based on our imaging of endogenously tagged ROCK relative to a biosensor for RhoA in *C. elegans* cortex, this delay is at most a few seconds, so it seems unlikely that it would affect the dynamics much. The parameters we used for RhoA and actomyosin reaction dynamics were obtained by fitting model predictions to observed time traces in the *C. elegans* cortex (Fig. 1b), so they effectively capture the experimentally observed time delays.

2. In the present study, the arrow from strain to Rho activation (Fig.1) arises from the dilution effect, \partial_u c (1+u) = 0, doesn't it? Could there be explicit local strain sensing via signalling?

Yes, the feedback from mechanical strain to RhoA activation arises from the dilution effect. It is certainly possible that there is explicit local strain sensing, but at present, we know of no such mechanism/signaling pathway operating in early *C. elegans* embryos. There is growing evidence in other contexts (i.e., not *C. elegans*) for strain sensing through direct binding of LIM domain proteins to strained actin filaments. However, not enough is known yet about these pathways to justify incorporating a more explicit form of strain sensing into our model. Instead, we chose to focus on an

indirect pathway for which there is strong existing experimental support, namely local F-actin-density dependent inhibition of RhoA (Bement et al, 2015, Michaux et al, 2018, Nishikawa et al, 2017, Landino et al, 2021, and others). This is actually a strain-rate sensing mechanism.

3. The flows that are discussed are cytoskeletal flows and not the cytosolic velocity that the system is embedded in. The cytoskeletal flows are therefore compressible. It would be good to clearly state this, as well as provide a justification for how you have ignored the cytosolic velocity field.

Following the reviewer's comment, we have now expanded our model to explicitly include cytosolic flows coupled to the cytoskeletal flows, where the cytoskeletal network is modelled as a porous viscoelastic gel. We show that the cytosol flow velocity can be eliminated to derive an effective equation for cytoskeletal flows, where the effect of cytosol is buried in a renormalized drag coefficient.

Does the dilution effect only apply to chemical species bound to the elastomer?

Yes, the unbound chemical species would just diffuse and exchange with bound species

4. The dynamical transition of the front propagation of Rho from stationary to moving to diffusive might be associated with different front propagation dynamical exponents, and may be analysed using ideas from Van Sarloos seminal work.

We thank the reviewer for this interesting comment. The front propagation analysis developed by Van Sarloos doesn't apply in the case studied in Fig. 4, since the unperturbed state (S=0) is in a linearly stable regime whereas those studied by Van Sarloos are linearly unstable to perturbations.

5. In equation 11, shouldn't the elastic response have both shear and compression? In which case the derivation of Eq. 11, would be more involved.

The model used here contains both shear and bulk elastic components, which through stress relaxation appear in the constitutive equation for the material stress tensor. This is now explained more clearly in the revised manuscript.

6. I did not see details of the numerical algorithm in the manuscript. Could this be provided please.

We have now included more details of the numerical algorithms used to solve the differential equations in the Methods section.
